# Comparative Study on the Corrosion Sensitivity and Microstructure of 15%SiCp/Al-Cu-Mg Aluminum Matrix Composites Under Different Aging Treatments

**DOI:** 10.3390/ma19091835

**Published:** 2026-04-29

**Authors:** Nan Guo, Zhiyong Li, Ran Pan, Yuansong Zeng, Pingan Xu, Yunhe Chang, Baosheng Liu

**Affiliations:** 1 AVIC Changhe Aircraft Industry (Group) Corporation Ltd., Jingdezhen 333002, China; 2 AVIC Manufacturing Technology Institute, Beijing 100024, China; 3 China Aeronautical Key Laboratory for Plastic Forming Technologies, Beijing 100024, China; 4 Beijing Key Laboratory of Digital Plastic Forming Technology and Equipment, Beijing 100024, China

**Keywords:** corrosion behavior, electrochemical impedance spectroscopy, 15%SiCp/Al-Cu-Mg, aging

## Abstract

A comparative investigation of the corrosion behavior evolution of 15%SiCp/Al-Cu-Mg aluminum matrix composites (AMC) subjected to different heat treatments in a salt spray environment containing 5wt% NaCl was performed. Metallographic microscopy was used to observe the surface morphology of the corroded materials. Field-emission transmission electron microscopy (TEM) and scanning electron microscopy (SEM) were used for microstructural evaluation and elemental analysis of the samples. Polarization curves and electrochemical impedance spectroscopy (EIS) were also employed to investigate the corrosion performance of the particle-reinforced aluminum matrix composites under different heat treatments. The test results indicate that, in addition to the influence of various grain boundary precipitates and electrochemical inhomogeneities between the precipitate-free zone (PFZ) and the aluminum matrix, differences in electrochemical properties between the SiC reinforcement particles and the aluminum alloy matrix are also a primary factor contributing to the corrosion of the aluminum-based composites in a 5wt% NaCl salt spray environment. Microstructural observations and electrochemical testing of AMC specimens at different corrosion stages indicate that under-aged samples exhibit relatively higher intergranular corrosion susceptibility. Under prolonged exposure to a salt spray environment, the over-aged specimen exhibited more pronounced galvanic corrosion phenomena, specifically, a significant decrease in Charge transfer resistance (R_ct_) values and an increase in CPE values. R_ct_ results indicate that naturally aged AMC exhibits higher corrosion resistance than artificially aged AMC. With increased salt spray corrosion time, varying degrees of crevice corrosion occurred at the Al–SiC interface in all heat-treated samples.

## 1. Introduction

Discontinuously reinforced aluminum matrix composites (AMCs) offer an optimal combination of mechanical strength, modulus, and density, and are generally considered candidates for aerospace load-bearing components, such as fighter aircraft ventral fins, blade sleeves, and rotor systems [1,2]. Aluminum structures used in aerospace are often subject to pitting corrosion, a degradation mechanism that reduces their reliability, toughness, and integrity. Like aluminum alloys, AMCs are very sensitive to pitting corrosion, and some microstructures exhibit limited resistance to localized corrosion. For AMCs, the corrosion behavior of the aluminum matrix is significantly affected by material preparation processes, heat-treatment conditions, and operating environments, making the application of AMCs in aerospace applications complicated. Pitting corrosion is very likely to occur in aircraft, especially those that operate in marine environments. Therefore, corrosion resistance must be considered when using aluminum matrix composite structures. Among the various corrosion modes, salt-spray-induced corrosion can cause a range of problems, especially at joints between components made of different materials. Therefore, it is essential to evaluate the corrosion resistance of particle-reinforced aluminum matrix composites.

As an aviation-grade high-strength aluminum alloy, the corrosion behavior of the Al-Cu-Mg (2XXX) alloy has been extensively studied by many researchers. A widely accepted view of its corrosion behavior is that electrochemical inhomogeneities exist between various intermetallic particles (IMPs) and the metal matrix [3,4,5,6]. Borg et al. [3] noted that localized corrosion around precipitates in 2024 aluminum alloy is a significant source of pitting corrosion. Hughes et al. [7] studied different aging precipitates in a 2024 aluminum alloy. They found that the potential difference between intermetallic compound particles and θ and S phases plays an important role in synergistic corrosion. Shao et al. [8] believed that intermetallic compound particles, mainly Al_2_Cu, Al_2_CuMg, and some Al-Cu-Fe-Mn (possibly Si), significantly affect the corrosion response of 2024 aluminum alloy. In addition, the chemical composition and width of the precipitation-free zone (PFZs) are also key factors in determining whether intergranular corrosion occurs. Research by Liu et al. [9] shows that, in addition to the precipitated phase at the grain boundaries, the potential difference between the precipitation-free zone (PFZ) and the aluminum matrix can also lead to anodic dissolution.

When it comes to particulate-reinforced aluminum-matrix composites, galvanic corrosion—arising from varying degrees of potential difference between reinforcing phases (such as SiC, Al_2_O_3_, TiC, and AlN) and the aluminum matrix—leads to an accelerated corrosion rate at the interface for the aluminum matrix and precipitated phases, which possess more negative potentials. Extensive research has already been conducted to investigate various types of particulate-reinforced aluminum-matrix composites systematically. The electrical conductivity of the reinforcing phase depends not only on the specific type of reinforcing material but also on its purity. For instance, variations in the purity of SiC reinforcing phases can cause their electrical conductivity to span a vast range, from 10^−5^ to 10^13^ Ω·m [10], and consequently result in markedly different tendencies toward galvanic corrosion in SiC particulate-reinforced aluminum-matrix composites. Thus, on the one hand, Monticelli et al. [11] and Greene et al. [12] observed that when SiCw/Al composites were immersed in seawater or solutions containing high concentrations of Cl^−^ ions, pitting corrosion frequently occurred in the vicinity of the SiC whiskers, providing evidence of galvanic corrosion. On the other hand, Aylor et al. [13] did not observe this phenomenon in their study regarding the corrosion behavior of SiCw/6061Al composites. Therefore, the presence or absence of galvanic corrosion between the reinforcing particles and the aluminum alloy matrix is contingent upon the type of reinforcement used and the specific fabrication process employed for the composite material. For example, during the fabrication of SiC-particulate-reinforced aluminum-matrix composites, the formation of Al_4_C_3_ may occur because the potential of Al_4_C_3_ is more negative than that of the aluminum alloy matrix, which leads to galvanic corrosion. Studies by Ahmad et al. [14] explicitly indicate that the SiC/Al interface serves as the preferential site for localized corrosion, resulting in a corrosion rate for the composite material that is two to three times higher than that of the unreinforced alloy. Similarly, Dikici et al. [15] confirmed that the incorporation of SiC reinforcing phases shifts the pitting potential to more negative values, thereby increasing corrosion susceptibility and resulting in a significantly higher corrosion rate for the composite material than for the unreinforced alloy. Additionally, the introduction of SiC particles further shifts the pitting potential toward the negative direction, thereby increasing the material’s overall susceptibility to corrosion.

Since a small amount of Si impurities inevitably exists in silicon carbide reinforcing particles, the Mg_2_Si formed by Si and magnesium can reduce the formation of S(Al_2_CuMg) and other Mg-related second-phase particles, and change the Cu/Mg ratio of solute in the aluminum matrix [16]. Some researchers [4,5] have found that the Cu/Mg ratio plays a crucial role in the aging response of Al-Cu-Mg alloys, because changes in this ratio can significantly affect the order and quantity of aging precipitates, thereby affecting the anodic dissolution process inside the material and the formation of passivation film near pitting sites. Since the potential (E_corr_) of the Mg_2_Si phase is approximately −1.16 V to −1.5 V [6,17], which is greater than the potential (E_corr_) of the S (Al_2_CuMg) phase (approximately −0.92 to −0.93 V) [18] and the potential (E_corr_) of the Al_2_Cu phase (approximately −0.63 to −0.69 V) [19], the changes in the order and quantity of precipitated phases in chloride solution will affect the corrosion resistance of SiCp/Al-Cu-Mg aluminum composite materials to a certain extent.

Furthermore, due to the presence of hard and brittle SiC particles, the ductility and fracture toughness of the quenched SiCp/Al composite material are worse than those of the unreinforced aluminum alloy [20]. Therefore, it is worthwhile to study how to produce particle-reinforced aluminum matrix composites with high strength, plasticity, and fracture toughness while maintaining corrosion resistance. Various heat treatments can improve the strength of aluminum matrix composites. Heat treatment is one of the most important methods for improving the mechanical properties of alloys, including solution heat treatment and aging treatment. Only a few studies have briefly explored the potential advantages of heat treatment in terms of mechanical properties, microstructure, and corrosion behavior.

Although various studies have investigated the effects of heat treatment on the mechanical and corrosion properties of Al-Cu-Mg alloys and composites, with some specifically focusing on the galvanic corrosion of aluminum-matrix composites [21], the underlying mechanisms by which different aging treatments influence the interfacial corrosion behavior of SiCp/Al-Cu-Mg composites remain unclear. Furthermore, only a limited number of studies have examined the evolutionary mechanisms of corrosion behavior in Al-Cu-Mg alloys under varying periods of corrosion exposure. Consequently, this paper aims to elucidate the impact of three distinct aging treatments on the corrosion resistance and mechanical properties of SiCp/Al-Cu-Mg aluminum-matrix composites. To accurately assess the evolutionary mechanisms of corrosion resistance in these composites, across different heat-treatment states and corrosion stages, this study systematically investigates the correlation between the microstructure and corrosion behavior of SiCp/Al-Cu-Mg composites through a comprehensive analysis involving hardness measurements, salt spray corrosion tests, surface morphology characterization, electrochemical impedance spectroscopy (EIS), and microstructural analysis.

## 2. Materials and Methods

In this study, the actual composition of the Al-Cu-Mg matrix in the 15%volSiCp/Al-Cu-Mg composite was Al-3.55Cu-1.35Mg-Fe0.06-Si0.24-Zn (<0.01) (wt.%). In the composite reinforced with 15% volumetric silicon carbide particles, the average particle size was 7 μm. The 15% vol SiCp/Al-Cu-Mg composite was provided by the Shenyang Institute of Metal Research, Chinese Academy of Sciences, and manufactured via powder metallurgy (PM). The samples required for each experiment were forged from 15% vol SiCp/Al-Cu-Mg composite forgings with a 60% deformation and dimensions of ∅ 250 mm × 80 mm, and then subjected to wire electrical discharge machining (EDM). This study used 85 mm × 40 mm × 5 mm aluminum-based composite salt spray corrosion test specimens, with the 85 mm × 40 mm surface perpendicular to the forging direction. This experimental plan aims to compare the mechanical properties and corrosion resistance of 15 vol.% SiCp/Al-Cu-Mg composites under three typical aging conditions: natural aging, under-aging, and over-aging. Furthermore, it plans to analyze the evolutionary patterns of the material’s corrosion behavior across these different heat-treated states by observing its corrosion response after various exposure durations, in conjunction with a series of electrochemical tests.

All 15%volSiCp/Al-Cu-Mg composite samples were solution-treated at 520 ± 5 °C for 1 h in an electric-heating furnace (Nabertherm GmbH, Bremen, Germany) and then water-quenched at 20 °C. Subsequently, the samples were subjected to different aging treatments: natural aging at ambient temperature for 7 days (NA), artificial aging at 180 °C for 2 h (SA2), and artificial aging at 180 °C for 24 h (SA24) via an electric-heating Oven (Nabertherm GmbH, Bremen, Germany). The experimental procedures for studying the effects of different heat-treatment regimes on the corrosion resistance of the 15% vol SiCp/Al-Cu-Mg composite material are detailed in Table 1.

The Brinell hardness test was used to evaluate the age-hardening behavior of aluminum matrix composites. Tests were conducted on a Brinell hardness tester with a load of 250 kgf and a holding time of 30 s (Shandong Laishi Automation Technology Co., Ltd, Laizhou, China). Five measurements were performed on aluminum-matrix composite samples subjected to different heat-treatment conditions, and the average value was used as the hardness evaluation standard.

Aluminum matrix composite parts are exposed to various environmental factors—such as oxygen, light, water vapor, and microorganisms—during storage and use, which can ultimately lead to material damage and even loss of usability. Salt spray corrosion testing is one method to accelerate the simulation of these influencing factors. Samples measuring 85 mm × 40 mm × 5 mm were mirror-polished, then immersed in acetone, ultrasonically cleaned with a metal degreaser for 5–10 min, ultrasonically cleaned with pure water, wiped with ethanol, and dried. A salt spray chamber conforming to ISO 9227 (Singleton, Scch) [22] was used. A 5 ± 0.5 wt% NaCl solution with a pH of 6.5–7.2 was sprayed onto 15% SiCp/Al-Cu-Mg composite material at room temperature using a discontinuous spray device (Q-LAB, Westlake, OH, USA). The temperature of the salt spray chamber was maintained at 25 °C. After 3 h, 24 h, 48 h, 96 h, and 192 h of salt spray exposure, the samples were washed with purified water, dried, and the corrosion on their surfaces was observed and evaluated. Each test was repeated three times, and statistical results were obtained. The electrochemically corroded surface was scanned with a scanning electron microscope (SEM, TESCAN MIRA 3, Brno, Czech Republic), and the chemical composition of the corrosion products was analyzed using energy-dispersive X-ray spectroscopy (EDS) to understand the corrosion morphology better. X-ray Diffraction Phase Analysis with Cu Kαradiation (Bruker AXS GmbH, Karlsruhe, Germany) was used to detected the phase composition of corrosion product.

While salt spray corrosion testing is the most widely used accelerated corrosion test for evaluating the corrosion resistance of aluminum alloys and aluminum-based composites, this method relies primarily on visual observation of surface corrosion, making it difficult to detect subtle differences. Considering the accuracy and reliability of the results, electrochemical methods such as polarization curves and impedance spectroscopy have received considerable attention. Electrochemical impedance spectroscopy, a reliable method for elucidating reaction mechanisms, has been widely used to study zone-corrosion processes [23].

At 25 °C, a three-electrode system with Pt as the auxiliary electrode and a saturated calomel electrode (SCE) as the reference electrode was used. After polishing and ethanol degradation, 15% vol SiCp/Al-Cu-Mg composite samples under different heat-treatment conditions were selected as the working electrodes. The other end of the sample was coated to seal the side surface except for the test surface. The working electrode was the experimentally exposed surface of the sample (10 mm × 10 mm), and the electrolyte was a 5 wt% NaCl solution. Dynamic potential scanning of the samples was performed using an electrochemical workstation (GAMRY Reference 600, Gamry Instruments, Warminster, PA, USA) at a scan rate of 1 mV/s. Polarization experiments were conducted at open-circuit potentials ranging from −1.1 V to 1.5 V. Impedance data were processed using Zview software (Version 2.0, Scribner Associates Inc., Southern Pines, NC, USA), and parameters were simulated using an equivalent circuit.

The degree of corrosion was assessed by recording the cross-section of the corroded specimens and measuring the maximum corrosion depth of a specific corrosion pit using an optical microscope. Transmission electron microscopy observations were performed on a JEM-2100F microscope at an operating voltage of 200 kV (JEOL Ltd, Tokyo, Japan). Samples used for TEM observation were cut from samples under different heat-treatment conditions and then ground into 0.7–0.8 mm thick foils. Several 3 mm diameter disks were punched from these foils and subsequently electropolished using a solution of hydrogen nitrate and methanol (volume ratio 1:4). The state and type of precipitates in the sample microstructure were observed and characterized using TEM and high-resolution transmission electron microscopy.

## 3. Results

### 3.1. Effect of Heat Treatment on Hardness of Silicon Carbide Particle-Reinforced Aluminum Matrix Composites

**Figure 1:**
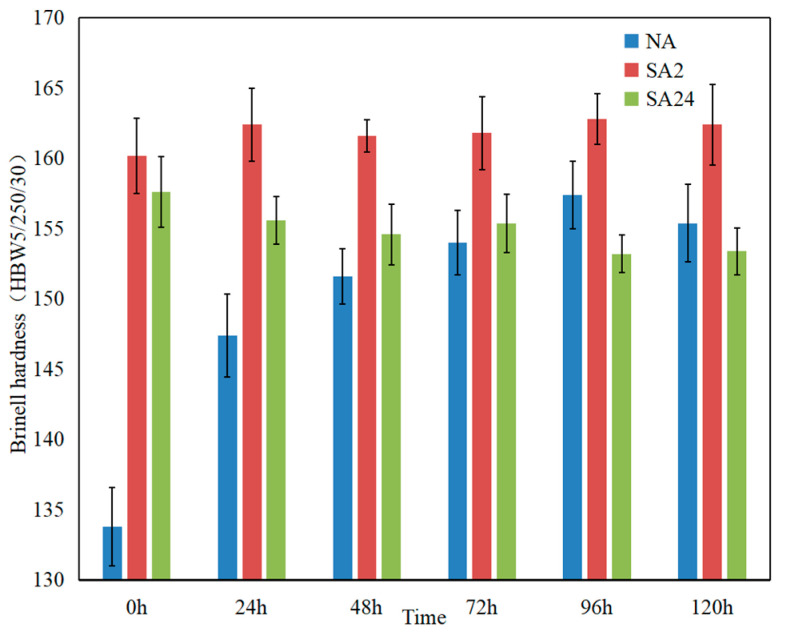
Changes in Brinell hardness of samples in three heat-treated states after natural aging for 1, 2, 3, 4, and 5 days.

Figure 1 shows the Brinell hardness of particle-reinforced aluminum matrix composite samples under different heat treatment states: samples under solution quenching and natural aging, samples aged at 180°C for 2 h after solution quenching, samples aged at 180°C for 24 h after solution quenching, and samples naturally aged for 1 day, 2 days, 3 days, 4 days, and 5 days after their respective heat treatments.

As shown in Figure 1, the hardness of the NA state samples significantly increases several days after solution treatment due to the precipitation of solute atoms from the supersaturated solid solution. This is due to the formation of fine precipitates, which are entirely or partially coherent and hinder dislocation movement, thereby increasing the material’s hardness. In contrast, the Brinell hardness values of the SA2 and SA24 aged aluminum matrix composite samples show relatively small changes during the “maturation” treatment several days after heat treatment. SA24 shows a slight decrease in Brinell hardness several days after heat treatment, perhaps because the size of the coarse precipitates continues to change slightly. It can be seen that the properties of all samples stabilize after 4–5 days. The hardness evolution directly mirrors the microstructural evolution induced by aging treatments, including the formation of GP zones in natural aging, fine needle-like θ′ precipitates in the under-aged state, and coarse θ/S phases in the over-aged state. These precipitates dominate both the age-hardening response and the corrosion susceptibility of SiCp/Al-Cu-Mg composites, as widely verified in Al-Cu-Mg alloy and its SiC-reinforced composites [24]. Hardness tends to be stable after 4–5 days of aging, indicating that the precipitation, size and distribution of second phases have reached a steady state. Only under a stable microstructure can subsequent corrosion test results be highly repeatable and comparable, which is of great significance for evaluating corrosion resistance and the engineering applications of materials.

### 3.2. Corrosion Behavior of Materials

After salt-spray corrosion treatment, corrosion bubbles began to adhere to the sample surface, indicating an increase in corrosion spots. Uniform corrosion gradually developed into intergranular corrosion and pitting, which could lead to spalling, blistering, and cracking. To characterize corrosion sites, the surface morphology of aluminum-based composite samples under different heat-treatment conditions was observed and analyzed using SEM after 3 h, 48 h, and 192 h of salt spray treatment.

**Figure 2:**
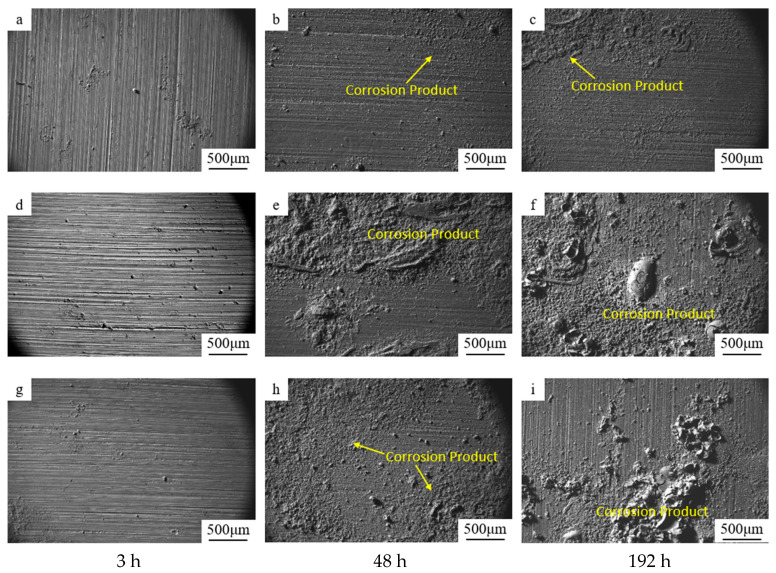
Corrosion morphologies of 15%volSiCp/Al-Cu-Mg composite having undergone different aging methods after experienced salt spray tests (5% NaCl solution) for 3 h, 48 h and 192 h. (**a**–**c**) NA state; (**d**–**f**) SA2; (**g**–**i**) SA24.

**Figure 3:**
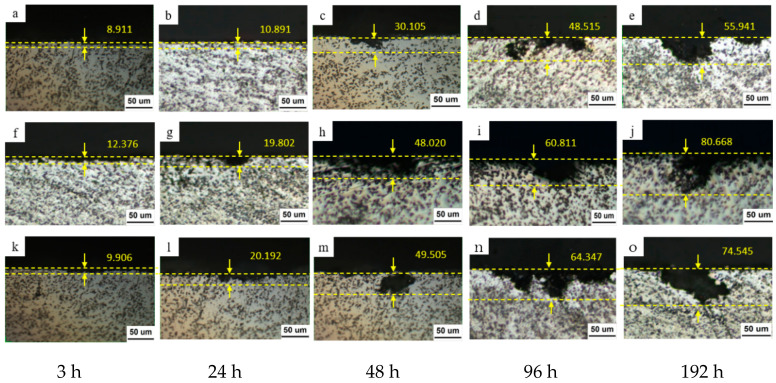
Surface morphology of 15%volSiCp/Al-Cu-Mg composite specimens corroded by salt spray atmosphere for 3 h/24 h/48 h/96 h/192 h of (**a**–**e**) NA state and (**f**–**j**) SA2 state and (**k**–**o**) SA24 state.

As shown in Figure 2, large-area spalling occurred on the sample surface after prolonged (>48 h) salt spray treatment, with a large amount of metal elements separating from the material surface. The surface morphology varied significantly with different aging and salt spray treatment times. For under-aged (180 °C × 2 h) and over-aged (180 °C × 24 h) samples, apparent delamination and numerous microbubbles appeared on the surface, as shown in Figure 2e,f,h,i. As shown in Figure 2b, the surface corrosion of the naturally aged sample was relatively slight after 48 h of salt spray treatment. After 192 h of salt spray treatment (Figure 2c), the surface corrosion was more severe, and the metal loss became significant, but still less than that of the sample aged at 180 °C. To investigate the degree of corrosion of the material under different heat-treatment conditions, the corrosion morphology across the sample thickness was characterized by metallographic microscopy; the micrographs are shown in Figure 3.

Using corrosion depth equivalence to assess the surface quality of materials and thus determine the degree of corrosion is an effective method. As shown in Figure 3 corrosion often develops toward the interior of the material along grain boundaries or at the interface between the reinforcing phase particles and the matrix. In general, for samples treated with solution quenching followed by 180 °C for 2 h, under the same salt spray treatment time, the area of corrosion on the material surface is deeper than that of samples treated with solution quenching followed by natural aging and samples treated with solution quenching followed by aging at 180 °C for 24 h. After solution quenching and aging at 180 °C for 2 h and 24 h, the maximum surface corrosion depths of the aluminum-based composite samples after 3 h of salt spray corrosion were 12.376 μm and 9.906 μm, respectively. After 48 h of salt spray corrosion, the corrosion depths increased to 48.020 μm and 49.505 μm, respectively. After 192 h of salt-spray corrosion, the corresponding surface corrosion depths were 80.668 μm and 74.545 μm, respectively.

In this study, after solution quenching and natural aging of 15% vol SiCp/Al-Cu-Mg in a 5 wt% NaCl salt spray environment for different times, the surface corrosion depth increased from 8.911 μm after 3 h of salt spray treatment to 55.941 μm after 192 h, which are 69.35% and 75.04% of the values of the SA2 and SA24-time aluminum-based composite samples under the same salt spray corrosion conditions, respectively. The corrosion resistance of the aluminum matrix composites studied in this paper is significantly affected by the heat-treatment method. The NA state sample showed the least corrosion, followed by the sample aged at 180 °C for 24 h. Intergranular corrosion and galvanic corrosion are two forms of material corrosion that occur under salt-spray conditions. During intergranular corrosion, corrosion products accumulate at grain boundaries, generating wedging forces that raise the material surface. That is to say, the greater the sensitivity of the metal matrix composite to intergranular corrosion and galvanic corrosion, the greater its sensitivity to salt spray corrosion. Obviously, the corrosion resistance of the aluminum matrix composite under the natural aging regime is higher than that of the SHT + 180 °C/2 h and SHT + 180 °C/24 h heat-treated states. Therefore, it can be inferred that, for particle-reinforced aluminum matrix composites, corrosion driven by the potential difference between the precipitated phase and the aluminum matrix still plays a dominant role in the material’s corrosion resistance, while pitting corrosion locations are relatively random. To further analyze the evolution of the surface microstructure of aluminum matrix composites during corrosion under salt spray conditions, the microstructure obtained by electrical scanning was analyzed by EDS.

**Figure 4:**
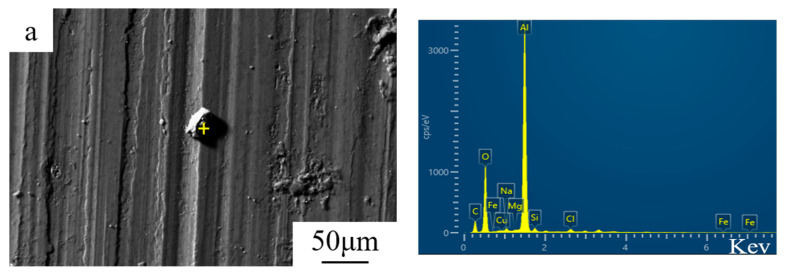

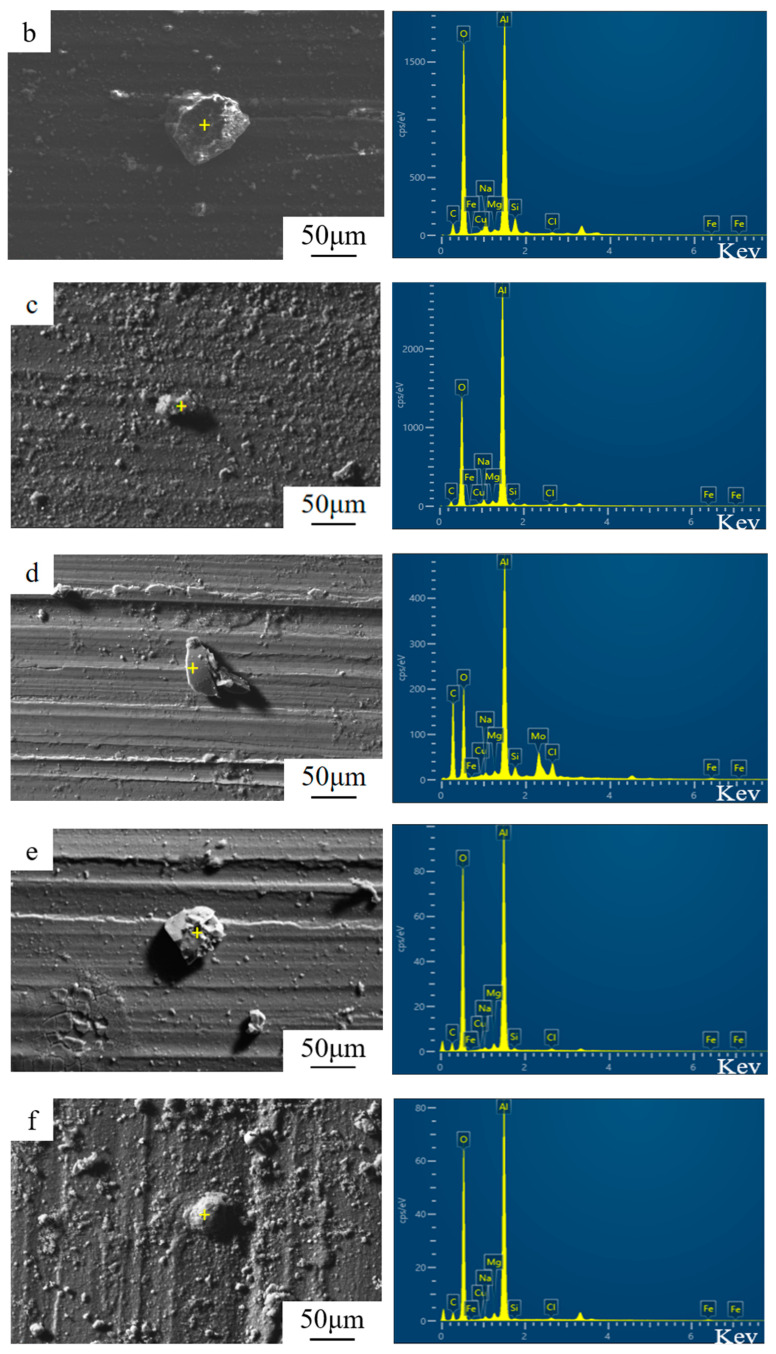

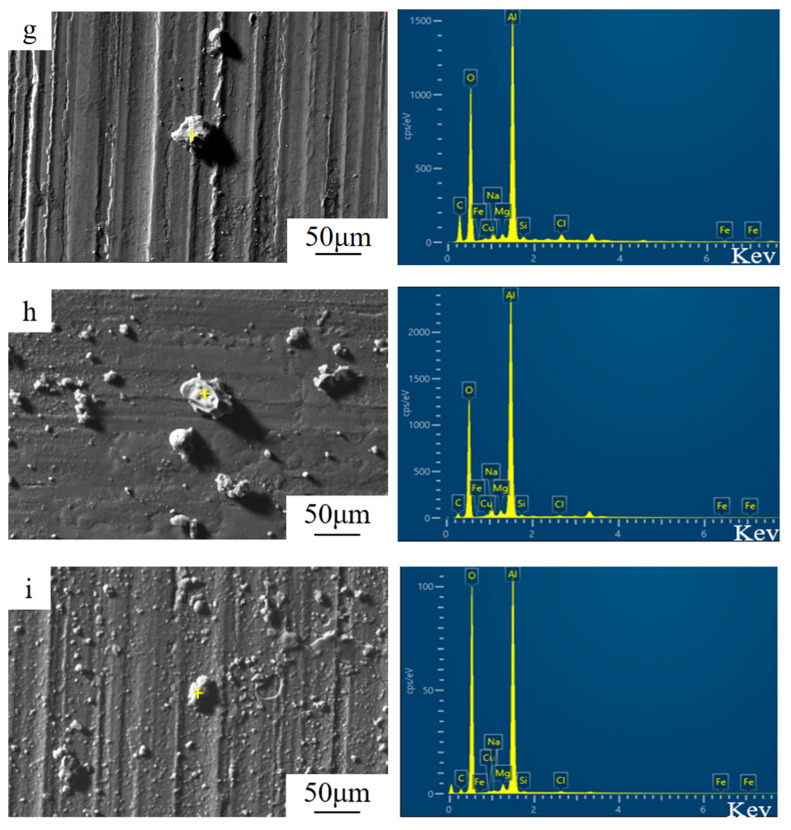
SEM and EDX analysis of the corrosion products in NA ((**a**) 3 h, (**b**) 48 h, (**c**) 192 h), SA2 ((**d**) 3 h, (**e**) 48 h, (**f**) 192 h), and SA24 ((**g**) 3 h, (**h**) 48 h, (**i**) 192 h) samples. (Yellow +: the EDS Detection Location).

Representative corrosion products from samples heat-treated under three conditions were observed after 3 h, 48 h, and 192 h of salt-spray corrosion. Their morphology and corresponding elemental scanning analysis results are shown in Figure 4a–i. It can be inferred that the corrosion products marked in Figure 4a,d contain Fe element. Comparing the atomic ratios in Table 2, the Si content varies significantly among different samples. No Si was detected in the corrosion products in Figure 4i, and a small amount of Si was detected in Figure 4b–e, suggesting the possible presence of SiC and Al_2_O_3_ phases in these samples. The high levels of Cu and Mg detected in the samples in Figure 4e,f,h,i are likely due to the copper- and magnesium-rich corrosion products covering the material surface.

The EDS results show that the corrosion products in all samples are rich in O, Cu, Mg, Si, C, and Al elements. The average atomic percentage of aluminum in the products was as high as 23.08%, while the average atomic percentage of oxygen was 52.67%, indicating that Al_2_O_3_ was the main component of the material surface. For samples NA, SA2, and SA24, which underwent salt spray treatment for 192 h, the atomic percentages of copper and magnesium in their corrosion products were 0.21% and 0.45%; 0.57% and 0.93%; and 0.25% and 0.99%, respectively, indicating that as exposure duration in the corrosive environment increases, the atomic content of both Cu and Mg in the corrosion products of samples in different heat-treated states gradually increases; furthermore, the extent of corrosion in the SA2 and SA24 samples was significantly greater than that in the NA state. The energy-dispersive spectrum further reveals that Na and Cl elements have diffused into the corrosion products of the SiC-particle-reinforced composite material following a period of salt spray exposure. Consequently, this demonstrates that the corrosion mechanism of the aluminum-matrix composite in a salt spray atmosphere involves the breakdown and penetration of the passivation layer (oxide layer) by chloride ions.

**Figure 5:**
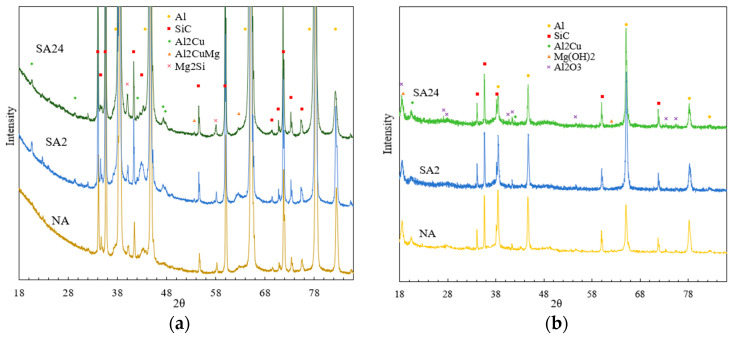
X-ray diffraction image of the 15%Vol SiCp/Al−Cu−Mg aluminum matrix composite. (**a**) Before corrosion; (**b**) Post corrosion (under salt spray treatment for 192 h).

To further analyze the nature of the corrosion products, X-ray diffraction (XRD) phase analysis was performed on the specimen surfaces before and after salt-spray corrosion testing. As shown in Figure 5, following the aging treatment, the 15% SiCp/Al-Cu-Mg aluminum-matrix composite exhibited precipitates such as Mg_2_Si, Al_2_Cu and Al_2_CuMg, in addition to the SiC and Al phases. After prolonged salt-spray corrosion, substances such as Al_2_O_3_, Al_2_Cu and Mg(OH)_2_ were detected on the material’s surface, indicating that because the corrosion potentials of most precipitates are higher than that of the aluminum matrix, they acted as anodes and dissolved.

For the 15%Vol SiCp/Al-Cu-Mg aluminum matrix composite shown in Figure 3, corrosion occurs at the interface between the reinforcing phase and the matrix, leading to peeling of the surface microstructure. Furthermore, as with aluminum alloys, corrosion occurs at grain boundaries without precipitate zones (see the fine cracks along the grain boundaries in Figure 4c,f,i), with corrosion extending parallel to the grain boundaries on the sample surface. Corrosion products thus accumulate at the grain boundaries. Since the precipitates inside the aluminum matrix composite sample are more uniformly distributed in the SA-state aluminum matrix composite after natural aging, intergranular corrosion can be mitigated, as further discussed in Section 4.1. Therefore, the interface between the reinforcing phase and the matrix, and the magnesium- and copper- depletion areas, are generally more likely to provide opportunities for intergranular salt spray corrosion attack.

### 3.3. Electrochemical Corrosion Test

**Figure 6:**
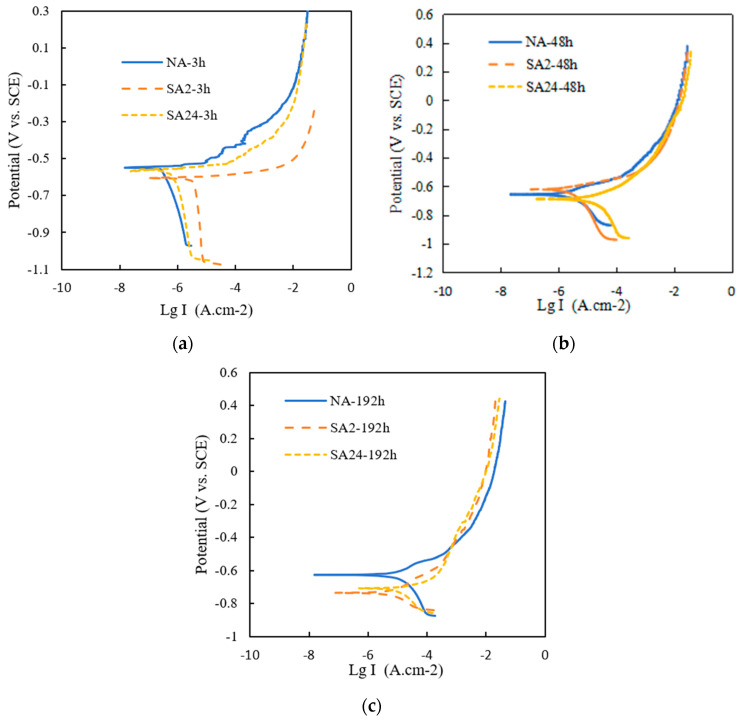
Polarization curves of the 15%volSiCp/Al−Cu−Mg with different heat treatments under salt spray treatment for (**a**) 3 h; (**b**) 48 h; and (**c**) 192 h.

Since corrosion is essentially an electrochemical process, the corrosion behavior of particle-reinforced aluminum matrix composites can be assessed by electrochemical testing. The polarization curves of aluminum matrix composites under different heat-treatment conditions are shown in Figure 6. Table 3 shows the electrochemical corrosion parameters obtained by Tafel extrapolation. The degree of corrosion can be reflected by comparing the corrosion potential and corrosion current. Although the corrosion potential (E_corr_) is the main driving force controlling the corrosion reaction and reveals the corrosion tendency, the corrosion current (i_corr_) is proportional to the corrosion erosion rate, so the i_corr_ of the aluminum matrix composites should also be compared. The cathodic region corresponds to the dissolution stage of the oxide film on the aluminum matrix composite surface and lies below the corrosion potential. The anodic region corresponds to the corrosion stage of the aluminum matrix composite itself and is above the corrosion potential. From the polarization curve morphology, it can be seen that the corrosion resistance of the aluminum matrix composite samples in all three heat-treatment states gradually weakens with increasing exposure time in the salt-spray corrosion atmosphere.

Under the same salt-spray conditions, the corrosion reactions of aluminum matrix composites in different aging states are similar. However, compared with the aged states SA2 and SA24, the polarization curve of the naturally aged NA state shows a significantly larger passivation region. The pitting potential corresponds to the location on the anodic polarization curve where the current density increases rapidly. Generally, the more positive the pitting potential, the less likely pitting corrosion will occur. The gradual slowdown in the rate of increase in current density as the potential continues to rise indicates that the reaction becomes diffusion-controlled. For both SA2 and SA24 samples, no obvious passivation region was observed in their anodic areas.

Besides corrosion current density and corrosion potential, polarization resistance is also an important criterion for judging the corrosion resistance of materials. The corresponding corrosion parameters, such as corrosion current density, corrosion potential, and polarization resistance, are shown in Table 3. Polarization resistance can be calculated using the following equation:

(1) $$R_{p}=\frac{∆E}{∆i}E_{corr}=\frac{\left|b_{a}\right|\cdot \left|b_{c}\right|}{2.3\left(\left|b_{a}\right|+\left|b_{c}\right|\right)}\cdot \frac{1}{I_{corr}}$$

where R_corr_ represents polarization resistance (kΩ⋅cm^2^), ΔE and Δi are polarization potential (mV) and polarization current (mA·cm^−2^), respectively, E_corr_ is the self-corrosion potential (mV), I_corr_ is the current density (mA·cm^−2^), and b_a_ and b_c_ represent the Tafel anode slope constant and the Tafel cathode slope constant, respectively. The polarization curves were fitted and analyzed using Zview software (Version 2.0). Important parameters such as the self-corrosion potential E_corr_, corrosion current density I_corr_, and corrosion resistance R_corr_ are shown in Table 3. A considerable R_corr_ value indicates that corrosion is less likely to occur.

For naturally aged NA aluminum-based composites, when the salt spray corrosion time increased from 3 h to 192 h, the E_corr_ value decreased from −550 mV to −626 mV, while the I_corr_ value gradually increased from 8.25 × 10^−5^ mA·cm^−2^ to 5.74 × 10^−4^ mA·cm^−2^. However, for SA2 and SA24 aluminum-based composite samples, when the salt spray corrosion time increased from 3 h to 192 h, the E_corr_ values decreased from −606 mV and −587 mV to −736 mV and −708 mV, respectively, while the I_corr_ values gradually increased from 4.69 × 10^−4^ mA·cm^−2^ and 3.18 × 10^−4^ mA·cm^−2^ to 1.61 × 10^−2^ mA·cm^−2^ and 1.22 × 10^−2^ mA·cm^−2^, respectively.

The polarization resistance also indicates that the NA aluminum matrix composite exhibits relatively better corrosion resistance, while the SA2 aluminum matrix composite shows corrosion resistance similar to that of the SA24 aluminum matrix composite. The electrochemical polarization results are broadly consistent with the corrosion levels of the samples in each heat-treated state shown in Figure 3. Therefore, compared to the aged aluminum matrix composite, the naturally aged aluminum matrix composite sample exhibits a lower corrosion current density and higher corrosion potential and polarization resistance, indicating that it offers relatively better resistance to salt spray corrosion. To systematically investigate the corrosion process of the materials, electrochemical impedance spectroscopy (EIS) was used for further analysis.

**Figure 7:**
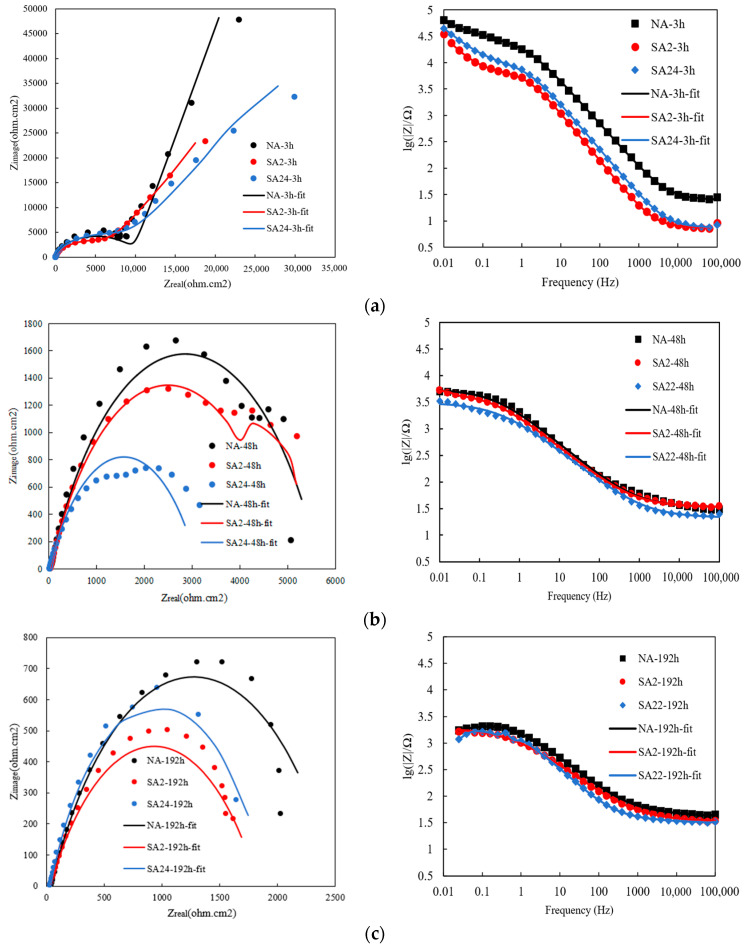
Electrochemical impedance spectroscopy (EIS) graphs of 15%volSiCp/Al-Cu-Mg under various heat treatments after tested in 5%NaCl solution for (**a**) 3 h; (**b**) 48 h; and (**c**) 192 h.

**Figure 8:**
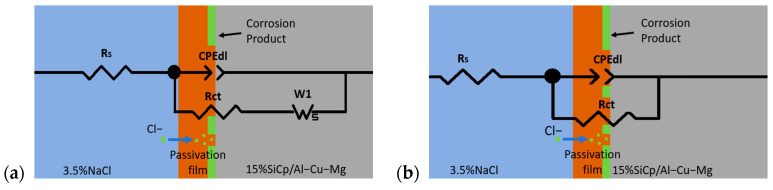
Equivalent electric circuit to fit the EIS data (**a**) for 3 h immersion and (**b**) for 48 h and 192 h immersions of 15%vol SiCp/Al−Cu−Mg in 5% NaCl solution. R_s_: solution resistance; CPE: origin surface double layer capacitive; R_ct_: charge transfer resistance.

Electrochemical impedance spectroscopy (EIS) is a non-destructive electrochemical technique used to characterize electrochemical reactions and corrosion product formation at the metal/electrolyte interface. Impedance spectra are typically displayed as Nyquist plots. To compare the salt spray corrosion susceptibility of the studied aluminum matrix composites under different aging treatments, Figure 7 shows the EIS plots for all samples. According to the Nyquist plot (Figure 7a), corrosion of the 15% vol SiCp/Al-Cu-Mg aluminum matrix composite began very early, with a small tail representing the diffusion process observed in the impedance spectrum of the sample after only 3 h of salt spray exposure. The EIS plot in the salt spray shows a capacitive arc in the high-frequency region, corresponding to the original surface of the aluminum matrix composite. In the low-frequency region, diffusion impedance appears, with its tilt angle deviating from approximately π/3 to π/4. For the aluminum matrix composite samples after 48 and 192 h of salt spray exposure, the Nyquist plots for all samples contained a semicircle. To better analyze the EIS results, a physical equivalent circuit was used to interpret the electrochemical impedance spectra. The fitted measurements were evaluated using chi-square values via Z-View software (Version 2.0). The recommended equivalent circuit is shown in Figure 8.

The solution resistance represents the ohmic resistance of the electrolyte between the working electrode and the reference electrode. Its variation reflects the accumulation or detachment of corrosion products on the material surface. The charge-transfer resistance is the most direct parameter for evaluating corrosion kinetics. It represents the difficulty of charge transfer across the electrode/electrolyte interface: a larger value indicates a slower corrosion rate, whereas a smaller value corresponds to accelerated corrosion. The constant phase element (CPE) is used to describe the non-ideal behavior of the electrical double layer, which deviates from pure capacitance due to surface roughness, current distribution inhomogeneity, and interfacial non-uniformity. W1 is the Warburg resistance Ws [25,26]. It appears in the low-frequency region when the corrosion process becomes diffusion-controlled, typically associated with oxygen reduction or the accumulation of corrosion products that hinder mass transfer. The impedance spectra in Figure 7 all contain a capacitance arc, indicating the presence of a time constant. An increase in the diameter of the capacitance arc indicates an increase in the impedance modulus. Table 4 shows the relevant measurements of various electrochemical parameters. A line representing the diffusion process exists in the Nyquist plot of the aluminum matrix composite after 3 h of exposure to salt spray; therefore, the Warburg resistance (W_s_) was added to the equivalent circuit. Warburg resistance is generally associated with the diffusion of corrosive ions to the surface. The Nyquist plots in the impedance spectra of the samples after 48 and 192 h of exposure to the salt-spray atmosphere have the same shape, indicating that the same corrosion mechanism may occur on the surface of the aluminum matrix composite samples under these corrosive conditions.

As shown in Table 4, the evolution of these EIS parameters directly reflects the progression of interfacial corrosion in the SiCp/Al-Cu-Mg composites. For the SA24 samples, Rct continuously decreased from 9963 Ω·cm^2^ at 3 h to 1516 Ω·cm^2^ at 192 h (an 84.6% reduction), demonstrating the enhanced electrochemical activity at the SiC/Al interface due to over-aging and indicating the formation of corrosion channels. Concurrently, the CPE coefficient Q increased from 4.54 × 10^−6^ mF·cm^−2^ to 1.46 × 10^−4^ mF·cm^−2^, reflecting the enlargement of effective surface area caused by interfacial corrosion pits and the detachment of SiC particles, consistent with the surface morphology observations in Figure 2 and Figure 3. The variation in solution resistance Rs (from 22.98 Ω·cm^2^ to 32.99 Ω·cm^2^ for SA24 samples) correlates with the accumulation of corrosion products such as Al_2_O_3_, as confirmed by the EDS analysis in Table 2.

Generally, the NA sample has a higher R_ct_ resistance than the SA2 and SA24 samples. Furthermore, the NA sample exhibited the lowest measured CPE, which may be related to the increased passivation layer thickness and the decreased dielectric constant of the oxide film. Systems with lower CPE and higher R_ct_ have higher impedance. The CPE value of the SA24 aluminum matrix composite sample, aged for 24 h after artificial aging, was lower than that of SA2 after 3 and 192 h of salt spray corrosion treatment, but slightly higher than that of SA2 after 48 h of salt spray corrosion treatment. These results further confirm that the NA sample has the highest corrosion resistance, which may be due to the relatively fine precipitates of the naturally aged aluminum matrix composite sample, which slow the gradual penetration of the corrosive solution. Overall, the EIS measurements show good agreement with the polarization results.

### 3.4. Microstructure Observation

**Figure 9:**
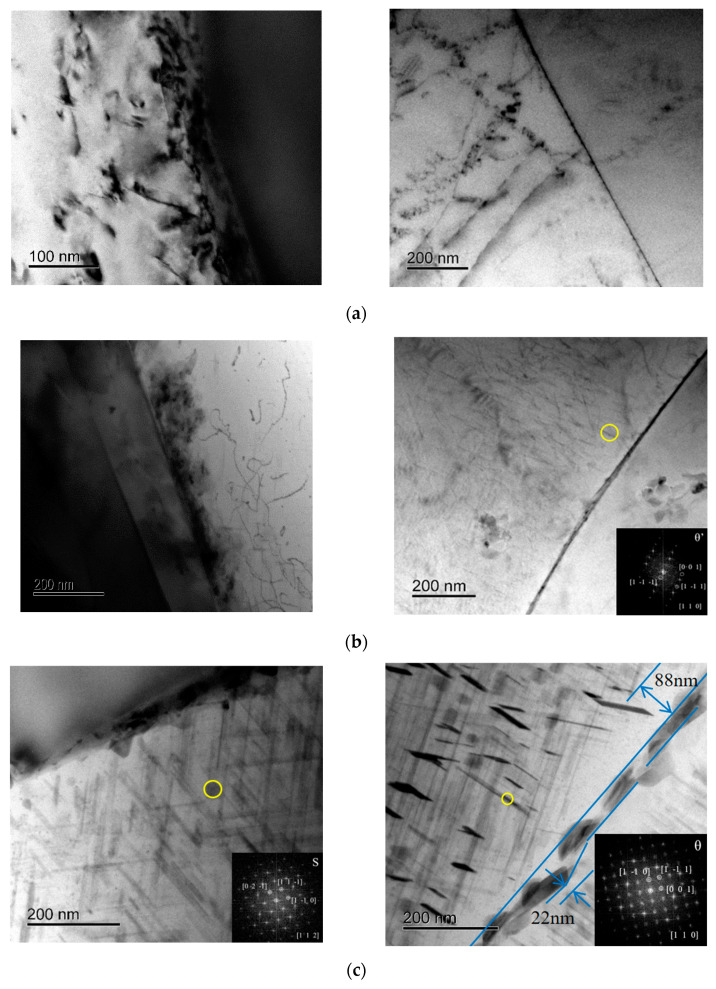
TEM transmission microstructure of 15%SiCp/Al−Cu−Mg aluminum composite samples under different heat treatment s; (**a**) NA state; (**b**) aging 180 °C × 2 h heat treatment (SA2 state); (**c**) aging 180 °C × 24 h heat treatment (SA24 state).

The strength/hardness and corrosion resistance of a material depend on its microstructure. Therefore, TEM observation was performed on 15%SiCp/Al-Cu-Mg aluminum matrix composites under different heat treatment states to characterize the initial microstructure of salt spray corrosion samples. Figure 9 shows bright-field TEM images of some of the studied samples. The precipitation sequence of 15%volSiCp/Al-Cu-Mg can be summarized as supersaturated solid solution (SSS) → GP region → metastable phase (θ′/S′) → stable phase (θ/S) [27,28]. The θ′ and S′ phases are the main strengthening phases of the material, semi-coherent with the matrix. Therefore, the precipitation and growth of the metastable phases is the reason for the increase in hardness [29,30,31]. Figure 9a shows a micrograph of 15%SiCp/Al-Cu-Mg aluminum matrix composites after natural aging at room temperature for 120 h. The GP region in the naturally aged (NA) sample shows a mottled appearance and slightly discrete contrast in the matrix. There are no precipitation-free zones at the grain boundaries. As shown in Figure 9b, after a short aging treatment of 2 h, the aluminum alloy matrix of the SA2 state aluminum matrix composite contains a large number of fine needle-like precipitates with a size of 20–30 nm, and continuous grain boundary precipitates exist at the grain boundaries. In contrast, when the material is in the over-aged heat-treated state (SA24), as shown in Figure 9c, the number of high-strength but unstable phases decreases. A large number of low-strength but stable coarse θ and S precipitates appear, whether inside the grains, at the grain boundaries, or at the interface between the reinforcing phase and the matrix. These precipitates consume a large number of surrounding solute atoms (Mg, Cu, etc.), and precipitation-free zones can be clearly distinguished at the grain boundaries.

## 4. Discussion

Many factors affect the corrosion resistance of particle-reinforced aluminum matrix composites. Common factors include grain size, recrystallization, matrix precipitates, grain boundary precipitates (GBP), precipitate-free zone width in the aluminum alloy matrix, and type, size, volume fraction, and morphology of the reinforcing phase. These factors usually work together to make the mechanism more complex. Compared with the aluminum matrix, grain boundary precipitates and non-precipitated phase zones can act as anodic phases, leading to electrochemical corrosion [32]. Under the combined action of corrosion and internal stress, intergranular cracks are more likely to propagate, thereby weakening corrosion resistance. Anodic phases usually dissolve preferentially. The extension of the aging environment can change the number, size, and spacing of grain boundary precipitates, thereby improving the corrosion resistance of long-time aged aluminum matrix composites [33,34]. However, our experimental results do not show that the corrosion resistance of over-aged aluminum matrix composites is the best. In addition, for reinforcing phase particles with significant differences in physical and electrochemical properties, such as silicon carbide particles, interfacial corrosion between the particles and the aluminum alloy matrix is also a key factor affecting the corrosion resistance of aluminum matrix composites. Therefore, this study explores the corrosion mechanism of particle-reinforced aluminum matrix composites in a long-term salt spray environment from two perspectives: interfacial reactions between grain boundary precipitates and non-precipitated phase zones, and reinforcing phase-matrix interactions.

### 4.1. Grain Boundary Precipitates and Precipitate-Free Zone

Grain boundaries are usually the main corrosion pathways for aluminum alloys. Generally, the corrosion potential of grain boundary precipitates is lower than that of the surrounding matrix [35]. During the corrosion process of aluminum alloys, grain boundary precipitates tend to become anodes and are preferentially corroded. Therefore, the rate of transition from pitting corrosion to intergranular corrosion (IGC) is affected by the continuity of grain boundary precipitates [36]. For aluminum matrix composites with Al-Cu-Mg as the matrix, the θ′ phase is a type of anodic phase and is usually distributed on the grain boundaries. When the material is under-aged or peak-aged in the heat treatment state, the θ′ phase is prone to form continuous corrosion channels along the grain boundaries, making the aluminum matrix composite exhibit poor corrosion resistance, such as SA2. As shown in Figure 9c, the SA24 aluminum-based composite material sample, after solution quenching and aging at 180 °C for 24 h, exhibits a wide fluctuation in the width of the precipitate-free zone, ranging from approximately 22 to 88 nm, generally smaller than the 90 nm and 100 nm values reported in the literature [37]. Simultaneously, it can be observed that the grain boundary precipitates are coarsened and exist in isolation, effectively blocking continuous corrosion channels at the grain boundaries, thereby enhancing the material’s corrosion resistance. Compared to the SA2 and SA24 samples, the NA sample has more G.P. zones, finer precipitates, and lower lattice distortion stress. Even after prolonged natural treatment, although solute atoms also form extremely fine precipitates at grain boundaries, the microstructure is more homogeneous than in other heat-treated states, reducing electrochemical differences within grains and between grain boundaries.

Therefore, this explains why, after 3 h of salt spray exposure, the SA24 aluminum-based composite material sample exhibits better electrochemical performance than the SA2 heat-treated state (Table 3 and Table 4), while the NA aluminum-based composite material sample shows better corrosion resistance after 3, 48, and 192 h of salt spray exposure. These expected results are consistent with previous findings [38,39]. However, when the SA24 sample was placed in a salt spray environment for 48 h, the discontinuously distributed grain boundary precipitates (see Figure 9c) did not appear to improve its electrochemical performance compared to NA or even SA2 state aluminum matrix composites; in fact, they were worse. When the SA24 sample was placed in a salt spray environment for 192 h, EIS (electrochemical impedance spectroscopy) and Tafel experimental data also showed that its corrosion resistance was similar to that of the SA2 sample but significantly weaker than that of the NA sample. To better understand the corrosion behavior of the SA24 aluminum matrix composite, the influence of the SiC reinforcing particles needs to be considered.

### 4.2. SiC Reinforcing Particles and Aluminum Alloy Matrix

In this study, the silicon carbide particles used in the material preparation were high-purity (99.9%) SiC particles, indicating that the SiC ceramic particles themselves should not react with the 5 wt% NaCl salt spray environment. However, the corrosion resistance of SiCp/Al composites in corrosive environments is usually lower than that of their corresponding unreinforced aluminum alloy matrix. In corrosive environments, the interface between the discontinuous reinforced phase and the aluminum alloy matrix is more prone to corrosion [40], forming microcracks and pores. Especially in chloride-containing media, interfacial corrosion exacerbates particle shedding [41], leading to crack propagation and a significant decrease in interfacial strength. As shown in Figure 9b,c, the shrinkage of the aluminum matrix after solution quenching results in significant stress and the accumulation of numerous dislocations and point defects at the interface between the aluminum matrix and the reinforcing phase. With prolonged aging, the high density of dislocations and point defects at the interface effectively promotes the precipitation of secondary phases. The size, quantity, and distribution of precipitates near the interface between the reinforced phase and the aluminum matrix in the over-aged SA24 sample are significantly higher than those in the SA2 sample. Therefore, the interface between the reinforced phase and the matrix in the over-aged SA24 sample is more prone to forming new corrosion microcells, thereby decreasing the material’s corrosion resistance.

Abdel Salam Hamdy et al. [42] investigated the influence of the interface of metal matrix composites on the corrosion susceptibility of materials and proposed that metal matrix composites exhibit different stages of corrosion mechanisms during corrosion in NaCl solution. Given that the volume fraction of the SiC reinforcing particles is approximately 15% and that they are distributed essentially uniformly within the aluminum alloy matrix, it is reasonable to infer the following: during short-term salt spray corrosion (3 h), although “galvanic corrosion”, arising from the galvanic coupling between the inert reinforcing phase (acting as the cathode) and the aluminum matrix (acting as the anode), has indeed occurred, its detrimental impact on the material’s corrosion resistance is relatively minor. This negative impact is less severe than that caused by the continuous grain boundary precipitates in the SA2 sample, which act as anodes to form corrosion channels [43], as illustrated in Figure 4d (where fine grain boundary corrosion cracks are already visible near the yellow dots). Therefore, in the early stages of corrosion, the SA24 material exhibits relatively good electrochemical performance than SA2.

**Figure 10:**
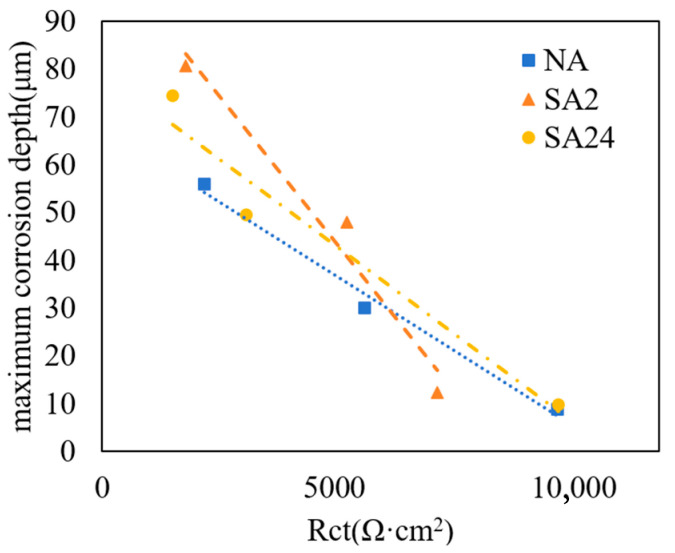
Correlation between Rct and maximum corrosion depth for different samples. (Dashed lines: fitting curves for samples under various conditions).

As the duration of salt spray corrosion increases, as illustrated in Figure 3, a substantial quantity of chloride ions penetrates the oxide layer, gradually eroding the substrate material. At this stage, the precipitates surrounding the silicon carbide (SiC) particles may undergo a phenomenon akin to the “intergranular corrosion” characteristic of the late stages of corrosion, as described by Abdel Salam Hamdy [42]; specifically, the SiC particles detach from the material surface, giving rise to “voids” that resemble pitting corrosion. Regarding this accelerated interfacial corrosion observed in over-aged SA24 specimens subjected to prolonged salt spray environments, Ahmad Z et al. [14] and Cui et al. [44] posit that the over-aging treatment leads to a significant increase in both the quantity and size of precipitates surrounding the SiC particles, thereby rendering the electrochemical corrosion at the interface more pronounced. Research [44] indicates that the corrosion current density of over-aged aluminum-matrix composites exceeds that of under-aged composites; this finding further substantiates the correlation between the increased volume of interfacial precipitates and the accelerated corrosion propagation. The measured values of Rcorr, Rs, Rct and the maximum corrosion depth, as shown in Figure 10, for the three distinct aging states examined in this study also lend support to this hypothesis. It is clear that this type of corrosion phenomenon occurs more frequently in SA24 samples than in SA2 samples, and its negative impact on the material’s corrosion resistance is more significant than in the early stages of corrosion. This explains why the corrosion resistance of SA24 samples decreased significantly after being placed in a salt spray environment for 48 and 192 h, and even fell below that of SA2 samples. A comparative analysis of the three aging conditions further reveals that the NA sample exhibits the highest Rct and lowest CPE values, corresponding to its finest and most uniformly distributed precipitates. The SA2 sample shows intermediate values, with continuous grain boundary precipitates forming initial corrosion channels. Under prolonged salt spray exposure, the SA24 sample exhibits the most pronounced Rct decline and CPE increase, directly reflecting accelerated corrosion propagation through widened SiC/Al interfacial corrosion channels induced by over-aging.

## 5. Conclusions

This paper investigated the corrosion behavior of 15% SiCp/Al-Cu-Mg aluminum-based composites subjected to three different heat treatments in a salt-spray environment using various methods. The main conclusions are as follows:

(1) From the corrosion potential, corrosion current, and electrical impedance spectra, it can be seen that after solution heat treatment and water quenching, the naturally aged (NA) aluminum-based composite samples exhibited lower corrosion sensitivity than the under-aged (SA2) and over-aged (SA24) samples. Compared with the underage sample (SA2), the over-aged sample (SA24) showed better corrosion resistance only under short-term salt-spray conditions.
(2) Scanning electron microscopy (SEM) and EDS chemical composition analysis revealed that the surface corrosion composition of the three heat-treated aluminum-based composites differed with increasing salt spray treatment time. The intergranular corrosion of the solution-quenched and under-aged sample was more severe than that of the naturally aged and over-aged samples, with relatively high levels of Cu and Mg in the corrosion products.
(3) The corrosion mechanism of over-aged particle-reinforced aluminum matrix composites under salt spray environment was speculated: In the early stage of corrosion, the galvanic corrosion between the particle-reinforced phase and the aluminum alloy matrix is limited, and the coarse, discontinuous grain boundary precipitates destroy the anodic corrosion channel, giving the material good corrosion resistance. However, as the corrosion time increases, the dense precipitates at the particle-reinforced phase interface in the over-aged aluminum matrix composite microstructure form a “corrosion channel” at the reinforcement–matrix interface, significantly weakening the material’s corrosion resistance.

## Figures and Tables

**Table 1:** Details of cryogenic thermal treatment scheme.

Material	Group Index	Temperature and Holding Time	Cooling Medium	Aging
15%SiCp/Al-Cu-Mg	NA	520 °C 60 min	Cold water	Ambient temperature 7 days
	SA2			180 °C/2 h
	SA24			180 °C/24 h

**Table 2:** Analysis of atomics ratio.

	O (%)	Cu (%)	Mg (%)	C (%)	Si (%)	Al (%)	Fe (%)	Na (%)	Cl (%)	Total (%)
NA-3 h	43.19	0.06	0.06	32.96	0.54	22.41	0.04	0.43	0.31	100
NA-48 h	60.02	0.16	0.22	18.87	1.44	17.08	0	2.14	0.07	100
NA-192	56.63	0.21	0.45	14.60	0.41	26.58	0	0.97	0.14	100
SA2-3 h	30.99	0.08	0.21	57.75	0.45	9.54	0.10	0.24	0.65	100
SA2-48 h	58.33	0.24	0.68	15.01	0.10	25.22	0	0.26	0.15	100
SA2-192	55.81	0.57	0.93	8.84	0.4	32.69	0	0.51	0.24	100
SA24-3 h	50.37	0.05	0.36	33.26	0.25	14.50	0.05	0.79	0.36	100
SA24-48 h	57.39	0.19	0.69	12.22	0.28	26.96	0	2.15	0.11	100
SA24-192	61.31	0.25	0.99	4.35	0	32.71	0	0.21	0.19	100

**Table 3:** Parameters of electrochemical polarization of 15%volSiCp/Al−Cu−Mg experienced various heat treatments under 5%NaCl salt spray atmosphere.

Samples	$E_{\mathrm{corr}\;}/(mV)$	$I_{\mathrm{corr}\;}/\left(mA\cdot {cm}^{-2}\right)$	$b_{a}/\left(mV\cdot {dec}^{-1}\right)$	$-b_{c}/\left(mV\cdot {dec}^{-1}\right)$	$R_{\mathrm{corr}\;}/\left(K\Omega \cdot {cm}^{2}\right)$
NA-3 h	−550	8.25 × 10^−5^	12.367	30.307	46.3
SA2-3 h	−606	4.69 × 10^−4^	4.577	3.462	7.4
SA24-3 h	−586	3.18 × 10^−4^	27.206	42.118	22.6
NA-48 h	−656	2.61 × 10^−4^	19.504	19.969	16.4
SA2-48 h	−621	4.91 × 10^−3^	82.890	564.880	6.4
SA24-48 h	−688	3.62 × 10^−4^	6.573	5.499	3.6
NA-192 h	−626	5.74 × 10^−4^	8.287	9.931	3.4
SA2-192 h	−736	1.61 × 10^−2^	34.301	36.408	3.1
SA24-192 h	−708	1.22 × 10^−2^	33.169	480.490	1.1

**Table 4:** Polarization curves fitting parameters of the studied aluminium composites in 5% NaCl solution.

Specimen	R_s_ (Ω⋅cm^2^)			CPE_dl_ (mF⋅cm^−2^)		
	3 h	48 h	192 h	3 h	48 h	192 h
NA	7.538	33.53	43.85	1.63 × 10^−5^	1.28 × 10^−4^	1.14 × 10^−4^
SA2	7.827	33.55	33.68	1.81 × 10^−5^	1.62 × 10^−4^	2.11 × 10^−4^
SA24	22.98	21.21	32.99	4.54 × 10^−6^	1.96 × 10^−4^	1.46 × 10^−4^
Specimen	R_ct_(Ω⋅cm^2^)					
	3 h		48 h		192 h	
NA	9808		5645		2208	
SA2	7221		5269		1792	
SA24	9963		3105		1516	

## Data Availability

The original contributions presented in this study are included in the article. Further inquiries can be directed to the corresponding author.
